# Does deep neuromuscular blockade during laparoscopy procedures change patient, surgical, and healthcare resource outcomes? A systematic review and meta-analysis of randomized controlled trials

**DOI:** 10.1371/journal.pone.0231452

**Published:** 2020-04-16

**Authors:** Amit D. Raval, Sohan Deshpande, Silvia Rabar, Maria Koufopoulou, Binod Neupane, Ike Iheanacho, Lori D. Bash, Jay Horrow, Thomas Fuchs-Buder

**Affiliations:** 1 Center for Observational and Real-world Evidence, Merck & Co., Inc., Kenilworth, NJ, United States of America; 2 Evidence, Modeling, and Synthesis, Evidera Inc., London, England, United Kingdom; 3 Department of Anesthesiology & Critical Care, Brabois University Hospital, University de Lorraine, CHRU Nancy, Vandoeuvre-les-Nancy, France; Cleveland Clinic, UNITED STATES

## Abstract

**Background:**

Deep neuromuscular blockade may facilitate the use of reduced insufflation pressure without compromising the surgical field of vision. The current evidence, which suggests improved surgical conditions compared with other levels of block during laparoscopic surgery, features significant heterogeneity. We examined surgical patient- and healthcare resource use-related outcomes of deep neuromuscular blockade compared with moderate neuromuscular blockade in adults undergoing laparoscopic surgery.

**Methods:**

We conducted a systematic literature review according to the quality standards recommended by the Cochrane Handbook for Systematic Reviews. Randomized controlled trials comparing outcomes of deep neuromuscular blockade and moderate neuromuscular blockade among adults undergoing laparoscopic surgeries were included. A random-effects model was used to conduct pair-wise meta-analyses.

**Results:**

The systematic literature review included 15 studies—only 13 were analyzable in the meta-analysis and none were judged to be at high risk of bias. Compared with moderate neuromuscular blockade, deep neuromuscular blockade was associated with improved surgical field of vision and higher vision quality scores. Also, deep neuromuscular blockade was associated with a reduction in the post-operative pain scores in the post-anesthesia care unit compared with moderate neuromuscular blockade, and there was no need for an increase in intra-abdominal pressure during the surgical procedures. There were minor savings on resource utilization, but no differences were seen in recovery in the post-anesthesia care unit or overall length of hospital stay with deep neuromuscular blockade.

**Conclusions:**

Deep neuromuscular blockade may aid the patient and physician surgical experience by improving certain patient outcomes, such as post-operative pain and improved surgical ratings, compared with moderate neuromuscular blockade. Heterogeneity in the pooled estimates suggests the need for better designed randomized controlled trials.

## Introduction

Deep neuromuscular block (dNMB) may facilitate the use of reduced insufflation pressure without compromising the surgical field of vision.[1, 2] However, its usage is limited due to a lack of predictable and rapid recovery with conventional neuromuscular block (NMB) antagonists (neostigmine) or spontaneous recovery. With the availability of selective relaxant binding agents—such as sugammadex for rocuronium/vecuronium bromide-induced NMB, which is uniquely indicated for the reversal of dNMB—rapid recovery can be achieved.

Bruintjes et al. and Park et al. reported surgical conditions and post-operative conditions related to the use of dNMB in laparoscopic procedures through a systematic review and meta-analysis of randomized controlled trials (RCTs) published up to December 2016 and October 2017, respectively. In both studies, dNMB showed improved surgical conditions when compared with other levels of block during laparoscopic surgery. However, pooled estimates had significant heterogeneity, suggesting the need for further studies accounting for these variations.[3, 4] Neither study included all peri-operative outcomes, such as need to change intra-abdominal pressure (IAP) during surgery, healthcare resource use-related outcomes, or patient-reported outcomes.[3, 4].

A systematic literature review (SLR) and meta-analyses were conducted to asses surgical patient- and healthcare resource use-related outcomes of dNMB compared with moderate neuromuscular blockade (mNMB) in adults undergoing laparoscopic surgery.

## Methods

The SLR was conducted in accordance with the quality standards recommended by the Preferred Reporting Items for Systematic Reviews and Meta-Analyses (PRISMA) statement and the Cochrane Handbook for Systematic Reviews.

### Criteria for inclusion in systematic review

We planned to include RCTs comparing outcomes of dNMB (defined as a train-of-four [TOF] count of zero or a post-tetanic count [PTC] count of 1–2) and mNMB (defined as a TOF count of 1–4) among adults undergoing laparoscopic surgeries. We excluded trials that reported a comparison of dNMB with no NMB, or shallow/restricted/standard NMB, and observational studies. We intended to capture all outcomes pertaining to surgical outcomes, post-operative patient-reported outcomes, and healthcare resource utilization. Outcomes relating to the quality of surgical field (#1) and the need to increase IAP levels (#2) were included to indicate any impact on the surgical procedure itself. The impact on the patient was assessed using measures of post-operative pain in the post-anesthesia care unit (PACU) (#3) and at 24 hours after surgery (#4), as measured by pain scores using an 11-point Likert scale (0 = no pain, 10 = worst possible pain); post-operative nausea/vomiting (#5) was also included. Duration of surgery (minutes; #6), length of hospital stay (days; #7), and length of recovery room stay (in minutes; #8) were included to consider the impact on healthcare resource utilization.

### Database search

The following electronic databases were searched (from inception to September 14, 2018) for publications relating to laparoscopic abdominal surgery in adults: Embase; MEDLINE, and MEDLINE In-Process via PubMed; Cochrane Library; the Cochrane Central Register of Controlled Trials; and the Database of Abstracts of Reviews of Effects. The searches were limited to publications in English, studies in humans, RCTs, and SLRs of RCTs only. No geographical restrictions were applied.

The search was conducted using the following keywords: “surgery,” “surgical operation,” “surgical procedure,” “laparoscopy,” “minimally invasive surgery,” “endoscopy,” “neuromuscular blocking agent,” “neuromuscular blocking,” “pneumoperitoneum,” “insufflation,” and “intra-abdominal pressure.” See S1 Table for full details of the search strings used.

A grey literature search was conducted for key conference proceedings from 2017 and 2018 (specifically, the American Society of Anesthesiologists, the European Society of Anesthesiology, and the International Anesthesia Research Society) and registries of ongoing clinical trials (clinicaltrials.gov; World Health Organization). The bibliographies of SLRs identified by the searches (published since 2016) were hand-searched for potentially relevant publications.

### Study selection

Two independent researchers screened publication abstracts against the study inclusion criteria described above; for publications that passed this first screening stage, publications were reviewed in full text. Any disagreements between the researchers were resolved by a third, senior investigator.

### Data extraction and risk-of-bias assessment

Data were extracted by one reviewer and validated by an independent reviewer in a standardized template developed a priori. The specific data elements extracted included study design, population baseline characteristics, interventions/comparators, and outcomes (including timing and definitions used). A quality assessment of the included studies was undertaken using the Cochrane Risk of Bias tool for RCTs,[5] with each included study with sufficient information (full publication) graded as being at low, moderate, or high risk of bias.

### Statistical analysis

Pair-wise meta-analyses based on a random-effects (RE) model were used to calculate pooled estimates and 95% confidence intervals (CI) for the relationships between levels of NMB and the outcomes of interest. For dichotomous variables, the effect size was expressed as a pooled odds ratio (pOR); for continuous variables, the effect size was expressed as a pooled mean difference (pMD) across studies if all studies used the same scale, or a pooled standardized mean difference (pSMD) if the scales used were different. A significance level of p = 0.05 was used for all variables. In each analysis, the heterogeneity of the estimates between studies was examined by computing I^2^ statistics, estimating between study variance (τ^2^) using restricted maximal likelihood (REML) methodology, and computing the p-value in the Cochrane’s Q test of homogeneity. The robustness of the study was explored using sensitivity analyses that included meta-analyses performed by a fixed-effects (FE) model and by serial single trial exclusions of studies causing heterogeneity. All analyses were performed using the “meta” package in R (version 3.5.1).

## Results

**Fig 1:**
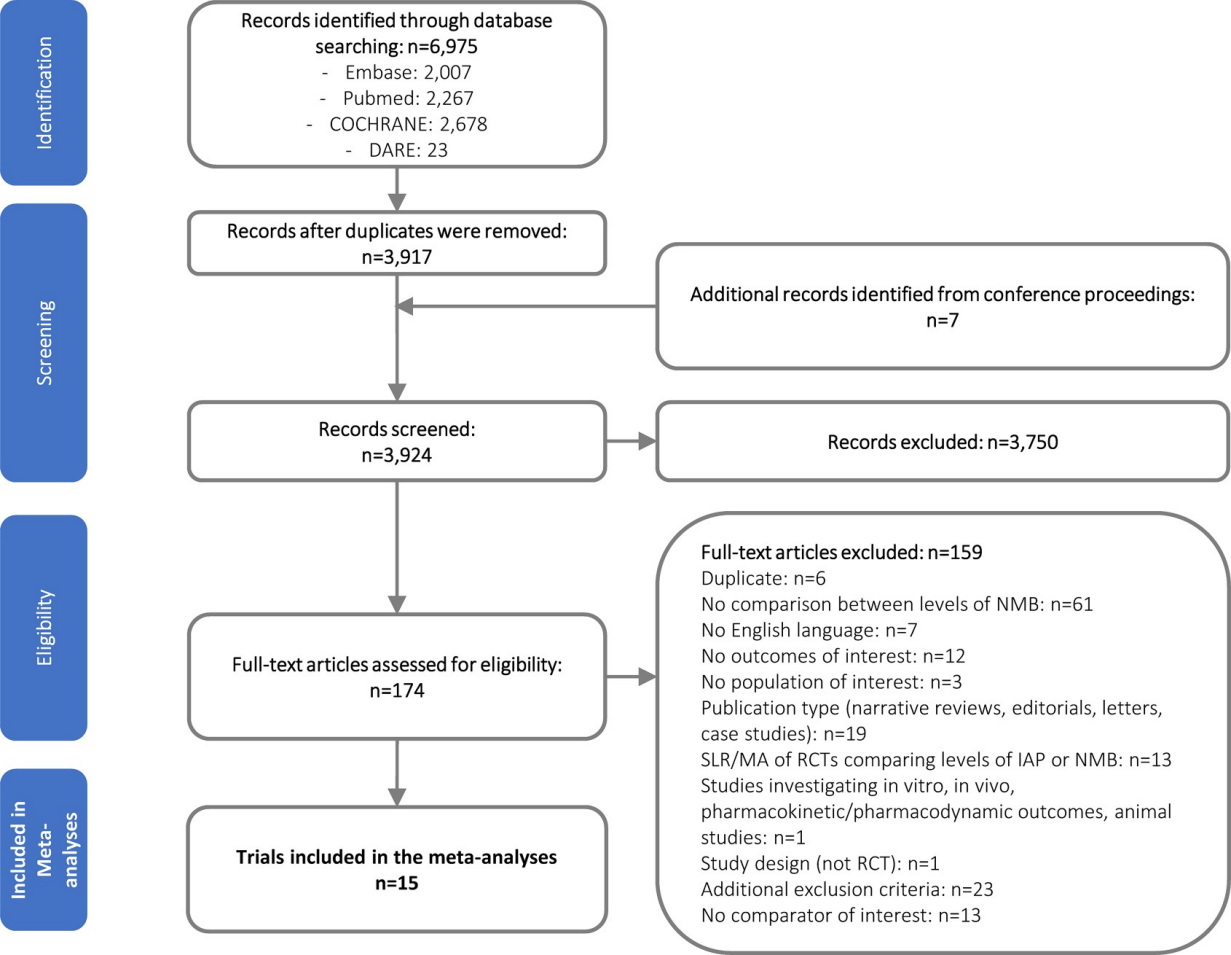
PRISMA diagram: Surgical, patient and HCRU outcomes from RCTs. Abbreviations: DARE: Database of Abstracts of Reviews of Effects; HCRU: healthcare resource utilization; IAP: intra-abdominal pressure; MA: meta-analyses; NMB: neuromuscular block; PRISMA: Preferred Reporting Items for Systematic Reviews and Meta-Analyses; RCT: randomized controlled trial; SLR: systematic literature review.

Of the 6,982 records identified (including records from grey literature), 15 fulfilled the study-selection criteria (Fig 1).

Table 1 describes the RCTs included in the SLR. Most were carried out in Asia (n = 6) or Europe (n = 7). The number of patients enrolled in each study ranged from 20 to 127. Table 2 describes the baseline characteristics of the study population in each RCT. The mean age across studies ranged from 41 to 63.9 years, and all trials enrolled patients with an American Society of Anesthesiologists (ASA) status ranging from I–III. Three studies enrolled patients with a high body mass index (≥40 kg/m^2^). Baseline characteristics were balanced between the intervention and comparator groups across all studies.

**Table 1:** Characteristics of included studies.

Study ID	Blinding Personnel	Laparoscopic Surgical Procedure	dNMB	mNMB	Country	N Randomized	N Completed
Torensma, 2016	A+S + N	Bariatric surgery	PTC 2–3	TOF 1–2	Netherlands	109	100
Zino, 2017	A	Bariatric surgery	PTC 0–1	TOF 1–2	US	107	107
Barrio, 2017	S	Cholecystectomy	PTC <5	TOF 1–3	Spain	90	90
Hojo, 2017	A	Cholecystectomy	PTC 1–2	TOF 1–2	Japan	60	60
Koo, 2016	A+P+S	Cholecystectomy	PTC 1–2	TOF 1–2	Republic of Korea	70	70
Rosenberg, 2017	A+P+S	Cholecystectomy	PTC 1–2	TOF 2–3	Austria, Finland, Germany, Italy, the UK	127	117
Kim, 2016	A+P+S	Colorectal resection	PTC 1–2	TOF 1–2	Republic of Korea	72	72
Koo, 2018	A+S	Colorectal surgery	PTC 1–2	TOF 1–2	Republic of Korea	70	64
Higaki, 2018	A	Gastrectomy	PTC 1–2	TOF 1–2	Japan	36	33
Baete, 2017	P + S	Gastric bypass	PTC 1–2	TOF 1–2	Belgium	60	60
Schmartz, 2016	S	Gastric bypass	PTC 1–5	TOF 1–4	France	69	69
Madsen, 2016	P+S+N+A	Hysterectomy	PTC 1–2	TOF 1–2	Denmark	110	99
Martini, 2014	S+P+A	Prostatectomy	PTC 1–2	TOF 1–2	Netherlands	24	24
Matsuzaki, 2018	-	Robotic prostatectomy	PTC 0–2	TOF 1–3	—	20	20
Yoo, 2015	P+A+S	Robotic prostatectomy	PTC 1–2	TOF 1–2	Republic of Korea	67	66

Abbreviations: dNMB: deep neuromuscular blockade; mNMB: moderate neuromuscular blockade; A: Assessors of Outcomes; N: Nurse; P: Patient; S: Surgeon; PTC: post-tetanic count; TOF: train-of-four; UK: United Kingdom; US: United States.

**Table 2:** Baseline characteristics of study population in the included studies.

Study ID	NMB Level	N at Baseline	Mean Age (SD) [years]	Male (%)	Mean BMI (SD) [kg/m^2^]	ASA Physical Status Classification (%)
Baete, 2017	dNMB	30	41 (13)	27	40.0 (3.0)	ASA I–III
	mNMB	30	42 (11)	13	41.0 (7.0)	ASA I–III
Barrio, 2017	dNMB	30	51.13 (10.13)	33.3	25.6 (3.3)	I: 33.3; II: 66.7
	mNMB	30	51.43 (10.28)	36.7	26.5 (3.0)	I: 30; II: 70
Kim, 2016	dNMB	30	57.1 (7.7)	60	23.0 (1.2)	I: 63.4; II: 33.3; III: 3.3
	mNMB	31	56.8 (9.6)	58.1	24.2 (1.3)	I: 51.6; II: 45.2; III: 3.2
Koo, 2016	dNMB	32	48.5 (28–67)	40.6	23.7 (2.4)	I: 72; II: 28
	mNMB	32	45.1 (27–68)	59.4	24.1 (3.4)	I: 75; II: 25
Koo, 2018	dNMB	32	60.0 (12.0)	62.5	24.0 (3.0)	I: 56; II: 44
	mNMB	32	58.0 (12.0)	59	24.0 (4.0)	I: 50; II: 50
Higaki, 2018	dNMB	16	-	-	-	-
	mNMB	17	-	-	-	-
Hojo, 2017	dNMB	-	-	-	-	-
	mNMB	-	-	-	-	-
Madsen, 2016	dNMB	55	47.0 (5.0)	0	23.1 (3.0)	I: 94.5; II: 5.5
	mNMB	55	48.0 (5.0)	0	24.2 (3.1)	I: 89.1; II: 10.9
Martini, 2014	dNMB	12	59 (28–74)	83.3	25.8 (3.2)	ASA II–III
	mNMB	12	60 (24–70)	83.3	25.9 (3.9)	ASA II–III
Matsuzaki, 2018	dNMB	5	-	100	-	ASA II–III
	mNMB	5	-	100	-	ASA II–III
	mNMB^§^	5	-	100	-	ASA II–III
Rosenberg, 2017	dNMB	36^£^	46.1 (17.7)	58.3	25.9 (2.9)	I: 41.7; II: 36.1; III: 5.6
		30	39.1 (13.6)	63.3	27.5 (4.0)	I: 53.3; II: 43.3; III: 3.3
	mNMB	31^£^	43.5 (15.6)	58.1	27.1 (3.7)	I: 54.8; II: 32.3; III: 9.7
		30	46.7 (13.8)	70	28.7 (2.7)	I: 56.7; II: 36.7; III: 6.7
Schmartz, 2016	dNMB	36	-	-	-	-
	mNMB	33	-	-	-	-
Torensma, 2016	dNMB	50	46.9 (10.6)	18	43.0 (4.5)	I: 0; II: 92; III: 8
	mNMB	50	47.2 (11.1)	22	43.3 (5.1)	I: 0; II: 84; III: 16
Yoo, 2015	dNMB	32	63.9 (6.1)	100	24.4 (2.5)	I: 25; II: 75
	mNMB	34	61.5 (5.4)	100	23.6 (2.0)	I: 38.2; II: 61.8
Zino, 2017	dNMB	27	-	-	47.4 (6.6)	-
	mNMB	27	-	-	47.1 (6.3)	-

^§^ study arm had peripheral nerve blockade^.^

^£^ study arm with low intra-abdominal pressure.

Abbreviations: ASA: American Society of Anesthesiology Physical Status Classification; BMI: body-mass index; dNMB: deep neuromuscular blockade; mNMB: moderate neuromuscular blockade; NMB = neuromuscular block; SD: standard deviation.

Although 15 studies met the inclusion criteria, only 13 were included in the meta-analysis, as two (Matsuzaki 2018[6], Schmartz 2016[7]) did not report any analyzable outcomes.

### Risk of bias assessment

**Fig 2:**
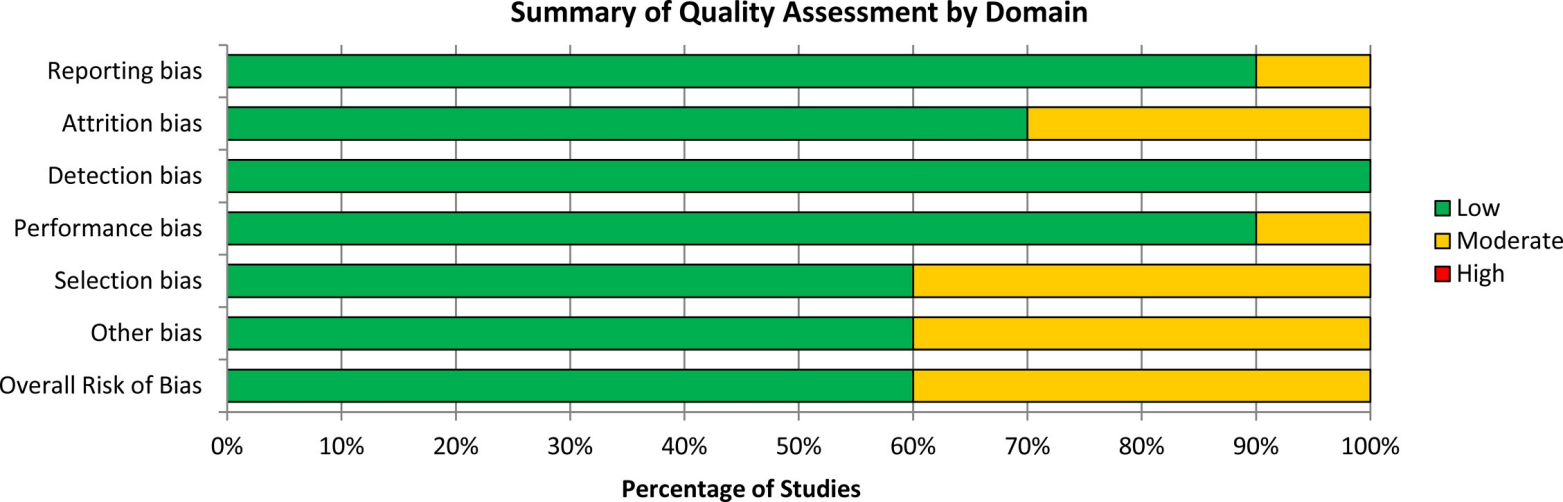
Summary of the quality assessment, via the cochrane risk of bias tool, for the identified RCTs reporting on NMBAs (quality assessment was possible for 10 studies for which full-text publications were available). Legend: The green, yellow and red colors in this figure respectively indicate low, moderate and high risk of bias. Abbreviations: NMBA: neuromuscular blocking agent; RCT: randomized controlled trial. For each domain, the percentage of studies deemed at low risk of bias was: reporting bias: 90%; attrition bias: 70%; detection bias: 100%; performance bias: 90%; selection bias: 60%; and other bias: 60%. None of the studies were deemed at high risk of bias for any domain.

Quality assessment via the Cochrane Risk of Bias tool was possible for the 10 studies for which full-text publications were available—of these, none were deemed to have a high risk of bias (Fig 2; 60% low risk [n = 6], 40% moderate risk [n = 4]). There was insufficient information available for the remaining five studies, since these were published as conference abstracts only.

### Surgical outcomes

#### Quality of surgical field

Five studies reported the quality of surgical field and were included in a meta-analysis (a total of six comparisons, as one study (Zino 2017[8]) reported results separately for two IAP populations). There was high heterogeneity across the studies (I^2^ = 77%; p<0.01). The outcome was measured subjectively using a 4-point rating scale (1 = excellent; 4 = poor; Hojo 2017[9]) or 5-point Leiden rating scale (1 = extremely poor; 5 = optimal; Baete 2017[10], Martini 2014[11], Torensma 2016[12], Zino 2017[8]). For a continuous outcome, if different studies used measurement scales in opposite directions, means in all treatment arms were expressed in the same direction before conducting meta-analyses (standard deviation [SD] does not require changing sign). For instance, if a study measured pain using a 0–4 scale, where a higher score means worse pain, and most other studies reported pain using scales where a higher score means less pain, then the mean in that study was calculated as 4 (maximum possible score) minus the observed mean in the study. If different studies used a different range of scales to measure the same outcome, the standardized mean difference (SMD) was calculated in all studies and meta-analyzed.

**Fig 3:**
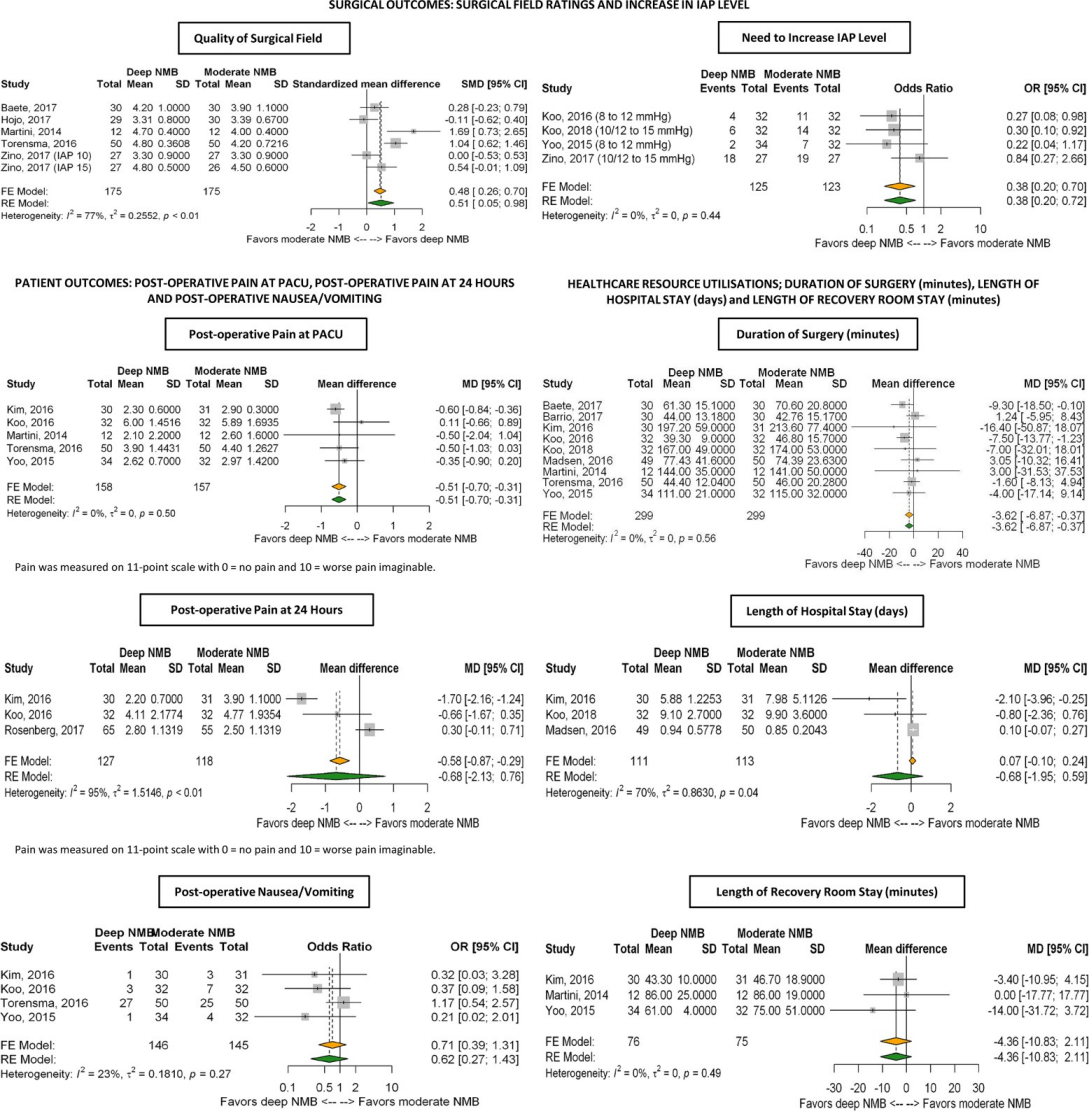
Meta-analysis results. Abbreviations: CI: confidence interval; FE: fixed effects; IAP: intra-abdominal pressure; MD: mean difference; NMB: neuromuscular block; OR: odds ratio; PACU: post-anesthesia care unit; RE: random effects; SD: standard deviation.

RE analyses showed that, compared with mNMB, dNMB was associated with a marginally statistically significant 0.5-point improvement in surgical field rating score: pSMD = 0.51 (95% CI: 0.05, 0.98; Fig 3).

#### Need to increase IAP level

Four studies reported on the need to increase IAP during surgical procedures and were included in this meta-analysis (Koo 2016[13], Yoo 2015[14], Koo 2018[15], Zino 2017[8]). RE analyses showed that the odds of this outcome were significantly reduced by 62% with dNMB compared with mNMB: pOR 0.38 (95% CI 0.20, 0,72; Fig 3). There was no heterogeneity across the studies (I^2^ = 0%; p = 0.44).

### Patient outcomes

#### Post-operative pain in the PACU

Five studies (Kim 2016[16], Koo 2016[13], Martini 2014[11], Yoo 2015[14], Torensma 2016[12]) reported on patient outcomes and were included in this meta-analysis. RE analyses showed that, compared with mNMB, dNMB reduced post-operative pain in the PACU by a modest 0.5-point improvement in pain scores: pSMD: –0.51 (95% CI: –0.70; –0.31; Fig 3). This reduction was observed as directionally consistent across different laparoscopic surgeries in subgroup RE analyses: colectomy (two studies) pSMD (95% CI): –0.34 (–1.01; 0.33); bariatric surgery (one study): pSMD (95% CI): –0.50 (–1.03; 0.03]; and prostatectomy (two studies): pSMD (95% CI): –0.37 (–0.88; 0.15). There was no heterogeneity across the studies (I^2^ = 0%; p = 0.50). In addition, when one study that reported only median data (Koo 2016[13]) was removed from the analysis, the resulting RE analysis was highly consistent with the main analysis: RE pSMD (95% CI): –0.55 (–0.75; –0.35).

#### Post-operative pain at 24 hours

Three studies reported on post-operative pain at 24 hours and were included in this meta-analysis (Kim 2016[16], Koo 2016[13], Rosenberg 2017[17]). RE analyses suggested there was no statistically significant difference between dNMB and mNMB for post-operative pain at 24 hours and also varied by surgical procedure (lower pain scores for colorectal surgery; higher pain scores for cholecystectomy). Given there was high heterogeneity between trials, the results could not be interpreted with any degree of certainty (I^2^ = 95%; p<0.01; Fig 3).

#### Post-operative nausea/vomiting

Four studies reported on post-operative nausea/vomiting and were included in this meta-analysis (Kim 2016[16], Koo 2016[13], Torensma 2016[12], Yoo 2015[14]). RE analyses showed no conclusive difference between dNMB and mNMB with regards to this outcome (pOR [95% CI]: 0.62 [0.27, 1.43]; Fig 3). There was little heterogeneity across the studies (I^2^ = 23%; p = 0.27).

### Healthcare resource utilization

#### Duration of surgery

Nine studies reported on duration of surgery and were included in this meta-analysis (Baete 2017[10], Torensma 2016[12], Barrio 2017[18], Kim 2016[16], Koo 2016[13], Koo 2018[15], Madsen 2016[19], Martini 2014[11], Yoo 2015[14]). RE analyses showed that, compared with mNMB, dNMB was associated with a modest (but statistically significant without heterogeneity, I^2^ = 0; p = 0.56) reduction in this outcome (pSMD [95% CI]: –3.62 minutes [–6.87; –0.37]; Fig 3).

#### Length of hospital stay

Three studies (Kim 2016[16], Koo 2018[15], Madsen 2016[19]) reported on the length of hospital stay and were included in this meta-analysis. RE analyses showed no conclusive difference between dNMB and mNMB for this outcome (pSMD [95% CI]: –0.68 days [–1.95, 0.59]; Fig 3). Heterogeneity was high between trials (I^2^ = 70%; p = 0.04).

#### Length of recovery room stay

Three studies (Kim 2016[16], Martini 2014[11], Yoo 2015[14]) reported on the length of recovery room stay and were included in this meta-analysis. RE analyses showed no conclusive difference between dNMB and mNMB on the time spent in the PACU (pSMD [95% CI]: –4.36 minutes [–10.83, 2.11]; Fig 3). There was no heterogeneity across the studies (I^2^ = 0%; p = 0.49).

## Discussion

Our study compared patient, surgical, and healthcare resource outcomes between dNMB and mNMB in adults undergoing laparoscopic surgery, with an aim to address key questions regarding the relative merits of the two approaches in this clinical setting. Findings suggest that compared with mNMB, dNMB was associated with improved surgical field of vision and higher vision quality scores; it should be noted there was considerable heterogeneity between studies for this outcome. In addition, dNMB was associated with a reduction in the post-operative pain scores in the PACU compared with mNMB (without any significant heterogeneity), as well as with greater consistency in maintaining the desired level of IAP without a need for an increase during the surgical procedures. With respect to resource utilization, we noted minor savings in surgical operation time (reduction of <5 minutes), but no differences in terms of recovery in the PACU or overall length of hospital stay with dNMB.

Our findings are broadly consistent with prior systematic reviews. Bruintjes et al. reported similar outcomes in favor of dNMB versus mNMB for improvements in surgical field of vision, reduced need to increase IAP during surgery, and improvements in post-operative pain in the PACU.[3] However, our study adds evidence with five other studies (Higaki 2018[20], Hojo 2017[9], Matsuzaki 2018[6], Schmartz 2016[7], Zino 2017[8]) beyond those included in the Bruintjes et al. analysis,[3] as well as robust methods with stricter criteria for consistently defining dNMB and mNMB.

Park et al. reported similar outcomes in favor of dNMB versus mNMB in terms of surgical field conditions and duration of surgery, while also observing—consistent with our analysis—no difference in terms of post-operative nausea and vomiting.[4] However, as well as including the five additional studies mentioned above, our analysis provided more comprehensive coverage of key outcomes, including reduced need to increase IAP levels during surgeries with dNMB. This is a more challenging endpoint to assess (due to the subjective nature of the rating scale)—in our study, it was associated with less heterogeneity across trials than vision-quality outcomes, thereby increasing confidence in the finding. This result may encourage future trials to capture this as a study endpoint.

A recently published RCT comparing dNMB and mNMB for patients undergoing gastric bypass surgery concluded that dNMB improves surgical conditions.[21] Due to the timing of the SLR and publication of this RCT, it was not included, though findings of our SLR are in line with its conclusion.

Thus, while evidence strongly suggests that dNMB improves surgical rating in certain procedures, less is known on the consequences of dNMB on patients’ outcome. The depth of NMB may affect patient outcome by two distinct mechanisms: improving surgical stillness and surgical conditions.

Sutton contractions or impaired visibility has been shown in a randomized trial in patients undergoing laparoscopic cholecystectomy (2 x 25 patients) with no NMB or dNMB.[22] The investigators assessed several sudden diaphragmatic or abdominal muscle contractions and instances with inadequate visibility at intrabdominal pressure at 15 mmHg. The total number of patients having these adverse events were 12/25 in the no NMB group; in one of them, a perforation of the liver capsule with the trocar following sudden diaphragmic movements, leading to important bleeding, occurred. In the dNMB group, adverse events were observed in 1/25 (p<0.001). Another randomized trial in patients undergoing laparoscopic hysterectomy (2 x 55 patients randomized to mNMB versus dNMB) found that 12/55 patients in the mNMB group experienced contractions in the diaphragm or abdominal muscles compared with 0/55 in the dNMB group (p<0.001[23]). The diaphragm is known to be significantly more resistant to non-depolarizing neuromuscular blocking agents than peripheral muscles, such as the adductor pollicis muscle typically used for intraoperative neuromuscular monitoring.[24] Deeper levels of NMB corresponding to a PTC 1–3 have shown to paralyze reliably the diaphragm and thus prevents sudden diaphragmatic or abdominal muscle movements.[25]

Of interest in this context, in a retrospective study, Boon et al. reported a significant reduction in unplanned 30-day readmission rates in retroperitoneal laparoscopic surgery in patients with an anesthesia protocol including high-dose rocuronium, and thus dNMB, compared with low-dose rocuronium. Better surgical conditions are supposed to explain these findings.[26] Similarly, Fuchs-Buder et al. recently demonstrated that switching from moderate to deep block improves surgical conditions, and poor surgical conditions were associated with a higher incidence of postoperative surgical complications.[21] Thus, increasing evidence suggests that dNMB not only improves surgical conditions, but better surgical conditions may lead to a better surgical outcomes.

The lack of association observed between the PACU stay and level of NMB block deserves further discussion. According to the findings from a Cochrane systematic review of RCTs, and consistent with the indicated use of reversal agents, sugammadex 4 mg/kg reversed dNMB (PTC 1–5) 45.78 minutes faster than neostigmine 0.07 mg/kg.[27] In the absence of sugammadex, which is uniquely indicated to reverse dNMB with its direct encapsulating mechanism of action, maintenance of dNMB through the end of surgery[28] would require prolonged intubation and mechanical ventilation (before reversal with neostigmine may be possible), which could lead to prolonged recovery room stay.

Before administration, because of their indirect mechanism of action, using cholinesterase inhibitors (e.g., neostigmine) to antagonize non-depolarizing NMB requires some degree of spontaneous recovery.[29, 30] While cholinesterase inhibitors decrease the enzymatic metabolism of acetylcholine and lead to an increase in acetylcholine—which competes with the non-depolarizing NMBA for the postsynaptic n-acetylcholine receptor, indirectly diminishing the effect the NMBA and facilitating neuromuscular recovery[29]—neostigmine-based reversal is limited to moderate and shallow NMB and doesn’t antagonize dNMB.[30, 31]

Similarly, five studies reporting the length of recovery room stay or length of hospital stays as outcomes utilized sugammadex as the reversal agent in the dNMB arms,[11, 14–16, 19] while two reported using neostigmine to reverse mNMB.[11, 15] Because sugammadex offers a rapid and predictable NMB recovery, even in deep block, it is possible that its impact on early NMB recovery resulted in observations in PACU length of stay that were not significantly different between dNMB and mNMB. Similarly, it is possible that if dNMB was maintained through the end of surgery and without the use of sugammadex, prolonged length of stay may be more common.

These analyses were robust in their use of consistent and rigorous definitions for dNMB and mNMB (including the exclusion of trials that used no NMB as the control arm) and the use of standardized scales for surgical ratings and SMD.

Our study is not without limitations. As with other SLRs, we noted the high degree of heterogeneity across trials for some outcomes (including quality of surgical vision and pain at 24 hours) and the limited number of studies available for some endpoints of interest. Therefore, findings should be interpreted in the context of these limitations. Due to the limited number of studies, we were not able to conduct all planned subgroup or sensitivity analyses. A majority of trials were industry sponsored. Also, our searches were limited to identify studies published in English only; however, we would have captured some studies in other languages that have English versions.

## Conclusion

Findings suggest that the use of dNMB may improve the patient and physician surgical experience by improving certain patient outcomes, such as post-operative pain and improved surgical ratings, compared with mNMB. However, to avoid post-operative residual paralysis and assure appropriate functioning of the upper airway once the patient is extubated (at recovery of the TOF ratio to >0.9, or even to unity) assessing neuromuscular recovery with acceleromyography is mandatory.[32–34]

Heterogeneity in the pooled estimates suggests the need for future, well-designed RCTs and standard definition of terms and rating scales to improve the precision of these findings and provide reliable data that can be used to inform clinical practice.

## Supporting information

S1 Checklist PRISMA 2009 checklist. (DOC)

S1 Table Search strategies. (DOCX)

## References

[1] Boon M, Martini C, Dahan A. Recent advances in neuromuscular block during anesthesia. F1000Res. 2018;7:167 10.12688/f1000research.13169.1 29497496 PMC5811671

[2] Brull SJ, Kopman AF. Current Status of Neuromuscular Reversal and Monitoring: Challenges and Opportunities. Anesthesiology. 2017;126(1):173–90. 10.1097/ALN.0000000000001409 27820709

[3] Bruintjes MH, van Helden EV, Braat AE, Dahan A, Scheffer GJ, van Laarhoven CJ, et al Deep neuromuscular block to optimize surgical space conditions during laparoscopic surgery: a systematic review and meta-analysis. Br J Anaesth. 2017;118(6):834–42. 10.1093/bja/aex116 28575335

[4] Park SK, Son YG, Yoo S, Lim T, Kim WH, Kim JT. Deep vs. moderate neuromuscular blockade during laparoscopic surgery: A systematic review and meta-analysis. Eur J Anaesthesiol. 2018;35(11):867–75. 10.1097/EJA.0000000000000884 30188357

[5] Higgins JPT, Green S. Cochrane handbook for systematic reviews of interventions version 5.1.0 [updated March 2011] 2011 [Available from: https://training.cochrane.org/handbook.

[6] Matsuzaki A, Noguchi S, Saito J, Nakai K, Kitayama M, Hirota K, editors. Poster No. 774: Efficacy of Abdominal Peripheral Nerve Block for Surgical Space Condition during Robot-Assisted Laparoscopic Surgery: A Pilot Study. International Anesthesia Research Society (IARS) Annual Meeting 2018; 2018; Chicago, IL, USA.

[7] Schmartz D, Brunaud L, Baumann C, Hilt L, Meistelman C, Fuchs-Buder T, editors. Does Deep Neuromuscular Blockade Improve Surgical Conditions in Patients Undergoing Gastric Bypass Surgery? American Society of Anesthesiologists (ASA) Annual Meeting 2016; 2016; Chicago, IL, USA.

[8] Zino A, Richter K, Del Porto-Dahms A, al. E, editors. Abstract No. A3091: Insufflation Pressure Not Depth of Neuromuscular Block Predicts Surgeon Satisfaction During Bariatric Surgery. American Society of Anesthesiologists (ASA) Annual Meeting 2017; 2017; Boston, MA, USA.

[9] Hojo T, Yamamoto H, Fukushima T, editors. Abstract No. A3071: Deep Neuromuscular Block Did Not Produce a Greater Improvement in Surgical Conditions in Laparoscopic Cholecystectomy than Moderate Neuromuscular Block: A Randomized Controlled Study. American Society of Anesthesiologists (ASA) Annual Meeting 2017; 2017; Boston, MA, USA.

[10] Baete S, Vercruysse G, ander Laenen M, De Vooght P, Van Melkebeek J, Dylst D, et al The Effect of Deep Versus Moderate Neuromuscular Block on Surgical Conditions and Postoperative Respiratory Function in Bariatric Laparoscopic Surgery: A Randomized, Double Blind Clinical Trial. Anesth Analg. 2017;124(5):1469–75. 10.1213/ANE.0000000000001801 28107276

[11] Martini CH, Boon M, Bevers RF, Aarts LP, Dahan A. Evaluation of surgical conditions during laparoscopic surgery in patients with moderate vs deep neuromuscular block. Br J Anaesth. 2014;112(3):498–505. 10.1093/bja/aet377 24240315

[12] Torensma B, Martini CH, Boon M, Olofsen E, In 't Veld B, Liem RS, et al Deep Neuromuscular Block Improves Surgical Conditions during Bariatric Surgery and Reduces Postoperative Pain: A Randomized Double Blind Controlled Trial. PLoS One. 2016;11(12):e0167907 10.1371/journal.pone.0167907 27936214 PMC5148011

[13] Koo BW, Oh AY, Seo KS, Han JW, Han HS, Yoon YS. Randomized Clinical Trial of Moderate Versus Deep Neuromuscular Block for Low-Pressure Pneumoperitoneum During Laparoscopic Cholecystectomy. World J Surg. 2016;40(12):2898–903. 10.1007/s00268-016-3633-8 27405749

[14] Yoo YC, Kim NY, Shin S, Choi YD, Hong JH, Kim CY, et al The Intraocular Pressure under Deep versus Moderate Neuromuscular Blockade during Low-Pressure Robot Assisted Laparoscopic Radical Prostatectomy in a Randomized Trial. PLoS One. 2015;10(8):e0135412 10.1371/journal.pone.0135412 26317357 PMC4552736

[15] Koo BW, Oh AY, Na HS, Lee HJ, Kang SB, Kim DW, et al Effects of depth of neuromuscular block on surgical conditions during laparoscopic colorectal surgery: a randomised controlled trial. Anaesthesia. 2018;73(9):1090–6. 10.1111/anae.14304 29727028

[16] Kim M, Lee K, KY. L, Min B, Yoo Y. Maintaining optimal surgical conditions with low insufflation pressures is possible with deep neuromuscular blockade during laparoscopic colorectal surgery: A prospective, randomized, double-blind, parallel-group clinical trial. Medicine (United States). 2016;95(9). 10.1097/MD.0000000000002920 26945393 PMC4782877

[17] Rosenberg J, Herring WJ, Blobner M, Mulier JP, Rahe-Meyer N, Woo T, et al Deep Neuromuscular Blockade Improves Laparoscopic Surgical Conditions: A Randomized, Controlled Study. Adv Ther. 2017;34(4):925–36. 10.1007/s12325-017-0495-x 28251555

[18] Barrio J, Errando CL, Garcia-Ramon J, Selles R, San Miguel G, Gallego J. Influence of depth of neuromuscular blockade on surgical conditions during low-pressure pneumoperitoneum laparoscopic cholecystectomy: A randomized blinded study. J Clin Anesth. 2017;42:26–30. 10.1016/j.jclinane.2017.08.005 28803124

[19] Madsen MV, Istre O, Staehr-Rye AK, Springborg HH, Rosenberg J, Lund J, et al Postoperative shoulder pain after laparoscopic hysterectomy with deep neuromuscular blockade and low-pressure pneumoperitoneum: A randomised controlled trial. Eur J Anaesthesiol. 2016;33(5):341–7. 10.1097/EJA.0000000000000360 26479510

[20] Higaki S, Hojo T, Fukushima T, Yamamoto H. Deep neuromuscular block failed to produce a greater improvement in the surgical conditions in a laparoscopic gastrectomy than moderate neuromuscular block: a randomized controlled study. General Anaesthesiology. 2018:18.

[21] Fuchs-Buder T, Schmartz D, Baumann C, Hilt L, Nomine-Criqui C, Meistelman C, et al Deep neuromuscular blockade improves surgical conditions during gastric bypass surgery for morbid obesity: A randomised controlled trial. Eur J Anaesthesiol. 2019. 10.1097/EJA.0000000000000996 30985536

[22] Blobner M, Frick CG, Stauble RB, Feussner H, Schaller SJ, Unterbuchner C, et al Neuromuscular blockade improves surgical conditions (NISCO). Surg Endosc. 2015;29(3):627–36. 10.1007/s00464-014-3711-7 25125097

[23] Madsen MV, Istre O, Springborg HH, Staehr-Rye AK, Rosenberg J, Lund J, et al Deep neuromuscular blockade and low insufflation pressure during laparoscopic hysterectomy. Dan Med J. 2017;64(5). 28552090

[24] Pansard JL, Chauvin M, Lebrault C, Gauneau P, Duvaldestin P. Effect of an intubating dose of succinylcholine and atracurium on the diaphragm and the adductor pollicis muscle in humans. Anesthesiology. 1987;67(3):326–30. 10.1097/00000542-198709000-00008 3631606

[25] Fernando PU, Viby-Mogensen J, Bonsu AK, Tamilarasan A, Muchhal KK, Lambourne A. Relationship between posttetanic count and response to carinal stimulation during vecuronium-induced neuromuscular blockade. Acta Anaesthesiol Scand. 1987;31(7):593–6. 10.1111/j.1399-6576.1987.tb02627.x 2891238

[26] Boon M, Martini C, Yang HK, Sen SS, Bevers R, Warle M, et al Impact of high- versus low-dose neuromuscular blocking agent administration on unplanned 30-day readmission rates in retroperitoneal laparoscopic surgery. PLoS One. 2018;13(5):e0197036 10.1371/journal.pone.0197036 29791482 PMC5965817

[27] Hristovska AM, Duch P, Allingstrup M, Afshari A. The comparative efficacy and safety of sugammadex and neostigmine in reversing neuromuscular blockade in adults. A Cochrane systematic review with meta-analysis and trial sequential analysis. Anaesthesia. 2018;73(5):631–41. 10.1111/anae.14160 29280475

[28] Jones RK, Caldwell JE, Brull SJ, Soto RG. Reversal of profound rocuronium-induced blockade with sugammadex: a randomized comparison with neostigmine. Anesthesiology. 2008;109(5):816–24. 10.1097/ALN.0b013e31818a3fee 18946293

[29] Naguib M, Flood P, McArdle JJ, Brenner HR. Advances in neurobiology of the neuromuscular junction: implications for the anesthesiologist. Anesthesiology. 2002;96(1):202–31. 10.1097/00000542-200201000-00035 11753022

[30] Plaud B, Debaene B, Donati F, Marty J. Residual paralysis after emergence from anesthesia. Anesthesiology. 2010;112(4):1013–22. 10.1097/ALN.0b013e3181cded07 20234315

[31] Fuchs-Buder T, Meistelman C, Alla F, Grandjean A, Wuthrich Y, Donati F. Antagonism of low degrees of atracurium-induced neuromuscular blockade: dose-effect relationship for neostigmine. Anesthesiology. 2010;112(1):34–40. 10.1097/ALN.0b013e3181c53863 19952724

[32] Capron F, Alla F, Hottier C, Meistelman C, Fuchs-Buder T. Can acceleromyography detect low levels of residual paralysis? A probability approach to detect a mechanomyographic train-of-four ratio of 0.9. Anesthesiology. 2004;100(5):1119–24. 10.1097/00000542-200405000-00013 15114208

[33] Eikermann M, Vogt FM, Herbstreit F, Vahid-Dastgerdi M, Zenge MO, Ochterbeck C, et al The predisposition to inspiratory upper airway collapse during partial neuromuscular blockade. Am J Respir Crit Care Med. 2007;175(1):9–15. 10.1164/rccm.200512-1862OC 17023729

[34] Eriksson LI, Sundman E, Olsson R, Nilsson L, Witt H, Ekberg O, et al Functional assessment of the pharynx at rest and during swallowing in partially paralyzed humans: simultaneous videomanometry and mechanomyography of awake human volunteers. Anesthesiology. 1997;87(5):1035–43. 10.1097/00000542-199711000-00005 9366453

