# Supplementary material for: Does deep neuromuscular blockade during laparoscopy procedures change patient, surgical, and healthcare resource outcomes? A systematic review and meta-analysis of randomized controlled trials
Source: PLoS One. 2020 Apr 16;15(4):e0231452. doi: 10.1371/journal.pone.0231452 (PMC7161978; doi:10.1371/journal.pone.0231452)
Supplement: S1 Table — (DOCX) [file pone.0231452.s002.docx]

**S1 Table. Search Strategies**

| **Search Number** | **Search Terms** | **Number of Hits** |
| --- | --- | --- |
| **EMBASE Search Strategy** | | |
| #1 | 'neuromuscular block*':ti,ab OR ((neuromuscul* NEXT/3 block*):ti,ab) OR 'neuromuscular blocking agent' OR 'neuromuscular blocking' | 19,870 |
| #2 | surgery:ti,ab OR surgical:ti,ab OR operation*:ti,ab OR 'surgical procedure*':ti,ab OR 'surgical condition*':ti,ab OR 'surgery'/exp | 5,328,315 |
| #3 | #1 AND #2 | 8,015 |
| #4 | 'pneumoperitoneum'/exp OR pneumoperitoneum:ti,ab OR 'insufflation'/exp OR insufflation:ti,ab OR 'intraabdominal pressure':ti,ab OR 'intra-abdominal pressure':ti,ab OR 'intra abdominal pressure':ti,ab | 31,009 |
| #5 | 'laparoscopy'/exp OR ((laparoscop*:ti,ab OR 'robot*':ti,ab) AND (surgery:ti,ab OR operation*:ti,ab OR 'surgical procedure*':ti,ab OR 'surgery'/exp)) OR 'minimally invasive surgery':ti,ab OR 'endoscopy'/exp OR endoscopy:ti,ab | 660,250 |
| #6 | #4 AND #5 | 11,269 |
| #7 | #3 OR #6 | 19,140 |
| #8 | 'randomized controlled trial'/exp OR 'randomized controlled trial'/exp/mj OR rct:ti,ab OR ((('randomized' OR 'randomised') NEXT/2 trial):ti,ab) OR (doubl* AND blind*:ti,ab) OR (singl* AND blind*:ti,ab) OR ('open':ab,ti AND label*:ti,ab) OR 'clinical trial':ti,ab | 856,344 |
| #9 | 'meta analysis'/exp OR 'systematic review (topic)'/exp OR 'meta analysis (topic)'/exp OR 'systematic review'/exp OR 'systematic and review' OR 'meta AND analysis' | 296,875 |
| #10 | #8 OR #9 | 1,118,144 |
| #11 | comment*:it OR editorial:it OR letter:it OR 'case report'/exp OR 'case study':ti,ab,de OR 'case studies':ti,ab,de | 3,851,882 |
| #12 | #7 AND #10 | 2,391 |
| #13 | #12 NOT #11 AND [humans]/lim AND [english]/lim | 2,007 |
| **PubMed Search Strategy** | | |
| #1 | neuromuscular block*[tiab] OR (neuromuscul*[tiab] AND block* [tiab]) OR Neuromuscular Blockade[MESH] OR “Neuromuscular Blocking Agents”[MESH] OR “neuromuscular blocking agent” OR “neuromuscular blocking” | 17,970 |
| #2 | surgery[tiab] OR surgical[tiab] OR operation*[tiab] OR surgical procedure*[tiab] OR surgical condition*[tiab] OR “Surgical Procedures, Operative”[MESH] | 3,901,483 |
| #3 | Search #1 AND #2 | 5,409 |
| #4 | (“Pneumoperitoneum, Artificial”[MESH] OR “Pneumoperitoneum”[MESH] OR pneumoperitoneum[tiab] OR “insufflation”[MESH] OR insufflation[tiab] OR “intraabdominal pressure”[tiab] OR “intra-abdominal pressure”[tiab] OR “intra abdominal pressure”[tiab]) | 17,435 |
| #5 | laparoscopy[MESH] OR laparoscopy[tiab] OR ((laparoscop*[tiab] OR robot*[tiab]) AND (surgery[tiab] OR operation*[tiab] OR surgical procedure*[tiab] OR “Surgical Procedures, Operative”[MESH])) OR “minimally invasive surgery”[tiab] OR “endoscopy”[MESH] OR  endoscopy[tiab] | 389,529 |
| #6 | #4 AND #5 | 6,973 |
| #7 | #3 OR #6 | 12,313 |
| #8 | “Randomized Controlled Trial” [Publication Type] OR “Randomized Controlled Trials as Topic” [Mesh] OR “randomized controlled trial”[tiab] OR “randomised controlled trial”[tiab] OR rct[tiab] OR ((randomized[tiab] OR randomised[tiab] OR random*[tiab]) AND trial* [tiab]) OR “clinical trial”[tiab] OR “Clinical Trial” [Publication Type] OR (doubl*[tiab] AND blind*[tiab]) OR (singl*[tiab] AND blind*[tiab]) OR (“open”[tiab] AND label*[tiab]) | 1,142,138 |
| #9 | (meta-analysis[Publication Type] OR "Meta-Analysis as Topic" [Mesh] OR meta analy*[tiab] OR (meta AND analysis) OR systematic review*[tiab] OR (systematic AND review)) | 386,572 |
| #10 | #8 OR #9 | 1,447,054 |
| #11 | comment [Publication Type] OR editorial [Publication Type] OR letter[Publication Type] OR Case Reports[Publication Type] | 3,352,651 |
| #12 | #7 AND #10 | 2,759 |
| #13 | #12 NOT #11 | 2,717 |
| #14 | #12 NOT #11 Filters: Humans | 2,520 |
| #15 | #12 NOT #11 Filters: Humans; English | 2,267 |
| **Cochrane Library Search Strategy** | | |
| #1 | (“neuromuscular block*”:ti,ab,kw OR (neuromuscul*:ti,ab,kw NEXT/3 block*:ti,ab,kw) OR “neuromuscular blocking” OR “neuromuscular blocking agent”) | 2,719 |
| #2 | MeSH descriptor: [Neuromuscular Blockade] explode all trees | 428 |
| #3 | MeSH descriptor: [Neuromuscular Blocking Agents] explode all trees | 1,367 |
| #4 | #1 OR #2 OR #3 | 3,162 |
| #5 | ((surgery:ti,ab,kw OR surgical:ti,ab,kw OR operation*:ti,ab,kw OR "surgical procedure*":ti,ab,kw OR "surgical condition*":ti,ab,kw)) | 182,217 |
| #6 | MeSH descriptor: [Surgical Procedures, Operative] explode all trees | 108,670 |
| #7 | #5 OR #6 | 221,552 |
| #8 | #4 AND #7 | 1,964 |
| #9 | ((pneumoperitoneum:ti,ab,kw OR insufflation:ti,ab,kw OR “intraabdominal pressure”:ti,ab,kw OR “intra-abdominal pressure”:ti,ab,kw OR “intra abdominal pressure”:ti,ab,kw)) | 2,074 |
| #10 | MeSH descriptor: [Pneumoperitoneum, Artificial] explode all trees | 279 |
| #11 | MeSH descriptor: [Pneumoperitoneum] explode all trees | 65 |
| #12 | MeSH descriptor: [Insufflation] explode all trees | 264 |
| #13 | #9 OR #10 OR #11 OR #12 | 2,074 |
| #14 | laparoscopy:ti,ab,kw OR ((laparoscop*:ti,ab,kw OR robot*:ti,ab,kw) AND (surgery:ti,ab,kw OR operation*:ti,ab,kw OR “surgical procedure*”:ti,ab,kw)) OR “minimally invasive surgery”:ti,ab,kw OR endoscopy:ti,ab,kw | 22,294 |
| #15 | MeSH descriptor: [Laparoscopy] explode all trees | 5,057 |
| #16 | (((laparoscop*:ti,ab,kw OR robot*:ti,ab,kw))) | 16,195 |
| #17 | MeSH descriptor: [Surgical Procedures, Operative] explode all trees | 108,670 |
| #18 | MeSH descriptor: [Endoscopy] explode all trees | 15,748 |
| #19 | #14 OR #15 OR (#16 AND #17) OR #18 | 29,554 |
| #20 | #13 AND #19 | 1,231 |
| #21 | #8 OR #20 | 3,118 |
| #22 | MeSH descriptor: [Randomized Controlled Trial] explode all trees | 137 |
| #23 | MeSH descriptor: [Randomized Controlled Trials as Topic] explode all trees | 13,527 |
| #24 | (meta-analysis:pt OR meta analy*:ti,ab,kw OR (meta AND analysis) OR systematic review*:ti,ab,kw) | 22,397 |
| #25 | “randomized controlled trial” OR “randomised controlled trial” OR rct OR ((randomized OR randomised OR random*) NEXT/3 trial*) OR “clinical trial” OR (doubl* AND blind*) OR (singl* AND blind*) OR (open AND label*) | 943,784 |
| #26 | MeSH descriptor: [Meta-Analysis] explode all trees | 0 |
| #27 | #22 OR #23 OR #24 OR #25 OR #26 | 945,368 |
| #28 | (comment OR editorial OR letter OR "case report*"):pt | 9,222 |
| #29 | #21 AND #27 | 2,694 |
| #30 | #29 NOT #28 | 2,678 |
| **DARE Search Strategy** | | |
| #1 | (neuromuscular block*) IN DARE | 29 |
| #2 | (neuromuscul* AND block*) IN DARE | 39 |
| #3 | (neuromuscular blocking agent) IN DARE | 3 |
| #4 | (neuromuscular blocking) IN DARE | 16 |
| #5 | MeSH DESCRIPTOR Neuromuscular Blockade EXPLODE 1 IN DARE | 9 |
| #6 | MeSH DESCRIPTOR Neuromuscular Blocking Agents EXPLODE 1 IN DARE | 6 |
| #7 | (surgery) IN DARE | 8,054 |
| #8 | (surgical) IN DARE | 4,684 |
| #9 | (operation*) IN DARE | 704 |
| #10 | (surgical procedure) IN DARE | 146 |
| #11 | (surgical condition) IN DARE | 0 |
| #12 | MeSH DESCRIPTOR Surgical Procedures, Operative EXPLODE 1 IN DARE | 139 |
| #13 | #1 OR #2 OR #3 OR #4 OR #5 OR #6 | 39 |
| #14 | #7 OR #8 OR #9 OR #10 OR #11 OR #12 | 9,633 |
| #15 | #13 AND #14 | 6 |
| #16 | MeSH DESCRIPTOR Pneumoperitoneum EXPLODE 1 IN DARE | 2 |
| #17 | (pneumoperitoneum) IN DARE | 17 |
| #18 | MeSH DESCRIPTOR insufflation EXPLODE 1 IN DARE | 19 |
| #19 | (insufflation) IN DARE | 47 |
| #20 | (intraabdominal pressure) IN DARE | 0 |
| #21 | (intra-abdominal pressure) IN DARE | 3 |
| #22 | (intra abdominal pressure) IN DARE | 3 |
| #23 | MeSH DESCRIPTOR laparoscopy EXPLODE 1 2 IN DARE | 623 |
| #24 | (laparoscopy) IN DARE | 718 |
| #25 | (minimally invasive surgery) IN DARE | 32 |
| #26 | (endoscopy) IN DARE | 582 |
| #27 | MeSH DESCRIPTOR endoscopy EXPLODE 1 2 IN DARE | 140 |
| #28 | (robot* AND surgery) IN DARE | 115 |
| #29 | (robot* AND procedure) IN DARE | 13 |
| #30 | (robot* AND operation) IN DARE | 14 |
| #31 | #16 OR #17 OR #18 OR #19 OR #20 OR #21 OR #22 | 60 |
| #32 | #23 OR #24 OR #25 OR #26 OR #27 OR #28 OR #29 OR #30 | 1,263 |
| #33 | #31 AND #32 | 17 |
| #34 | #15 OR #33 | 23 |
